# Sequential treatment with zoledronic acid followed by teriparatide or vice versa increases bone mineral density and bone strength in ovariectomized rats

**DOI:** 10.1016/j.bonr.2017.06.001

**Published:** 2017-08-19

**Authors:** T. Shimizu, T. Tanaka, T. Kobayashi, I. Kudo, M. Nakatsugawa, A. Takakura, R. Takao-Kawabata, T. Ishizuya

**Affiliations:** Pharmaceuticals Research Center, Asahi Kasei Pharma Corporation, 632-1 Mifuku, Izunokuni, Shizuoka 410-2321, Japan

**Keywords:** Zoledronic acid, Teriparatide, Sequential treatment, Ovariectomized rat, Bone mineral density, Bone metabolism

## Abstract

Bisphosphonates (BPs) and teriparatide (TPTD) are both effective treatments for osteoporosis, but BP treatment prior to daily TPTD treatment has been shown to impair the effect of TPTD in some clinical studies. In contrast, the loss of bone mineral density (BMD) that occurs after withdrawal of TPTD can be prevented by BP treatment. Although various studies have investigated the combination and/or sequential use of BP and TPTD, there have been no clinical studies investigating sequential treatment with zoledronic acid (ZOL) and TPTD (or vice versa). In this study, we evaluated the effects of sequential treatment with TPTD followed by ZOL, and ZOL followed by TPTD, using ovariectomized (OVX) rats.

Two months after OVX, osteopenic rats were treated with ZOL, TPTD, or vehicle for a period of 4 months (first treatment period), and then the treatments were switched and administered for another 4 months (second treatment period).

The group treated with ZOL followed by TPTD showed an immediate increase in BMD of the proximal tibia and greater BMD and bone strength of the lumbar vertebral body, femoral diaphysis, and proximal femur than the group treated with ZOL followed by vehicle. Serum osteocalcin, a marker of bone formation, increased rapidly after switching to TPTD from ZOL.

The group treated with TPTD followed by ZOL did not lose BMD in the proximal tibia after TPTD was stopped, while the group treated with TPTD followed by vehicle did lose BMD. The BMD and bone strength of the lumbar vertebral body, femoral diaphysis, and proximal femur were greater in the group treated with TPTD followed by ZOL than in the group treated with TPTD followed by vehicle. The increase in serum osteocalcin and urinary CTX after withdrawal of TPTD was prevented by the switch from TPTD to ZOL.

In conclusion, our results demonstrate that switching from ZOL to TPTD resulted in a non-attenuated anabolic response in the lumbar spine and femur of OVX rats. In addition, switching from TPTD to ZOL caused BMD to be maintained or further increased. If these results can be reproduced in a clinical setting, the sequential use of ZOL followed by TPTD or vice versa in the treatment of osteoporosis patients would contribute to increases in BMD that, hopefully, would translate into a corresponding decrease in the incidence of vertebral and non-vertebral fractures.

## Highlights

• Sequential treatment of zoledronic acid followed by teriparatide immediately increased bone mineral density.
• Sequential treatment of zoledronic acid followed by teriparatide immediately increased bone formation.
• Sequential treatment of teriparatide followed by zoledronic acid prevented the decrease of bone mineral density.
• Sequential treatment of teriparatide followed by zoledronic acid prevented the increase of bone resorption.

## 1 Introduction

Bisphosphonates (BPs) and parathyroid hormone (PTH) are antiresorptive and bone anabolic agents, respectively, and both are clinically effective treatments for osteoporosis. However, treatment with a combination of BP and PTH is not necessarily more effective than monotherapy with either one. For example, the increases in bone mineral density (BMD) at the spine and the femoral neck after combined use of daily teriparatide (TPTD; the 1–34 fragment of PTH) and oral alendronate (ALN; a commonly used BP) were smaller than after treatment with TPTD alone in postmenopausal women and in men (Finkelstein et al., 2010, Finkelstein et al., 2003). Moreover, combining PTH (1–84) with daily oral ALN has been reported to inhibit PTH-induced increases in volumetric BMD in women with postmenopausal osteoporosis (Black et al., 2003). Some reports have indicated that BP treatment prior to daily TPTD treatment impaired the effects of TPTD. In postmenopausal women who switched from BP to TPTD treatment, the increases in lumbar spine BMD occurred later, and decreases in proximal femur BMD occurred sooner, in those who were previously treated with daily oral ALN than in those who were previously treated with raloxifene. The effects on BMD of switching to TPTD after treatment with risedronate (RIS) were similar to the effects seen in women previously treated with ALN (Cosman, 2014) (Boonen et al., 2008) (Obermayer-Pietsch et al., 2008). Such effects are likely attributable to BP-related inhibition of bone formation, which is coupled to bone resorption.

Zoledronic acid (ZOL) is a BP that is administered once-yearly by intravenous infusion. Among BP agents, ZOL causes the greatest reduction in the vertebral fracture rate in osteoporotic patients (Zhou et al., 2016). As with other BP agents, ZOL causes BMD increases of up to 12% for as long as 6 years after administration (Black et al., 2012). In contrast to the combined effects of ALN and TPTD, the combination of ZOL and daily TPTD has been shown to result in earlier increases in BMD at the vertebral bodies and femoral neck after treatment initiation than monotherapy with TPTD (Cosman et al., 2011). In addition, the combination of TPTD and ZOL has been shown to be more effective than TPTD or ZOL therapy alone in immobilized, osteopenic rats (Vegger et al., 2014). These results suggest that different BP agents, when given in combination with TPTD, may cause different outcomes. While treatment with ZOL followed by TPTD is expected to be effective, there have been no clinical studies that verify the effects of this treatment protocol.

BMD is known to decrease after the termination of TPTD treatment if no subsequent antiresorptive drug therapy is given. Several studies have reported that switching to a BP effectively inhibits such decreases (Kaufman et al., 2005, Kurland et al., 2004, Sugimoto et al., 2013, Prince et al., 2005); however, ZOL was not the BP used in those studies, and no clinical studies have assessed the sequential use of TPTD followed by ZOL. The only report available is a non-clinical study that assessed the efficacy of sequential use of PTH (1–84) followed by ZOL in ovariectomized rats (Rhee et al., 2004); however, in that study the ZOL was administered once per week, and thus the results may not be applicable to the characteristic once-yearly regimen of ZOL used in humans.

In the present study, we used ovariectomized (OVX) rats to assess the effects on bone strength and bone turnover of sequential treatment with ZOL and TPTD using an administration protocol designed to mimic as closely as possible the clinical use of these drugs in humans.

## 2 Materials and methods

### 2.1 Animals and experimental design

Two hundred and ten 3-month-old female Sprague-Dawley rats (Charles River, Kanagawa, Japan) were purchased for this study and maintained until 6 months of age to allow them to become skeletally mature. The rats were housed in a dedicated laboratory animal facility with a 12-/12-h light/dark cycle and unrestricted access to tap water and food (CRF-1; standard diet of rats, Oriental Yeast, Tokyo, Japan). All experimental protocols were approved by the Experimental Animal Ethics Committee at Asahi Kasei Pharma Corp. and conducted in accordance with established guidelines concerning the management and handling of experimental animals.

**Fig. 1:**
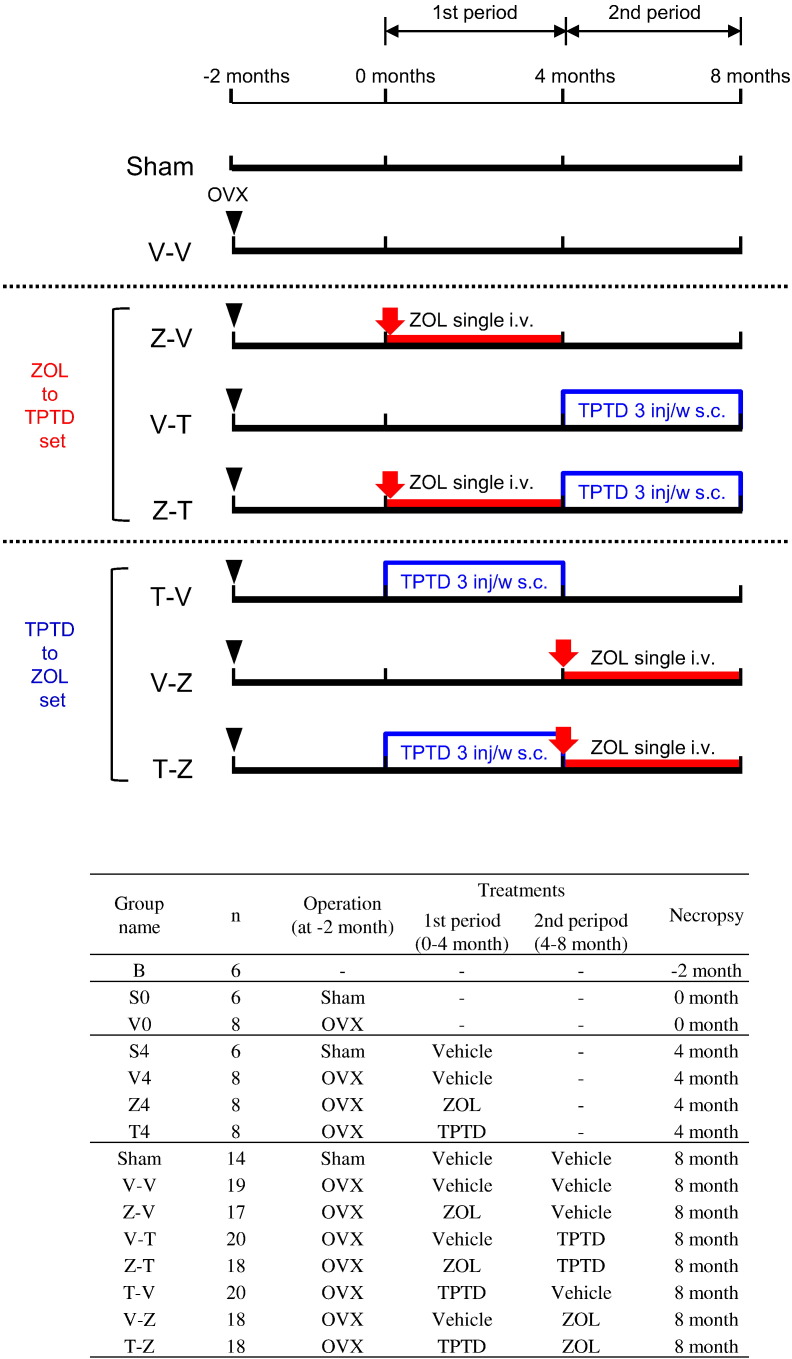
Experimental groups. ZOL to TPTD set: the effects of Z-T sequential treatment were compared with those of the Z-V or V-T therapy. TPTD to ZOL set: the effects of T-Z sequential treatment were compared with those of the T-V or V-Z therapy. All treatments were started (at month 0) 2 months after the OVX operation (at month − 2). 100 μg/kg ZOL was administered as a single intravenous injection. TPTD was administered subcutaneously at 6 μg/kg three times weekly. After 4 months of treatment with one of the drugs or vehicle, the animals started treatment with the other drug or vehicle. Animals in the satellite groups for each treatment (groups B through T4) were sacrificed at months − 2, 0, and 4 to harvest bone tissues. All groups and the treatments they underwent are shown in the chart at the bottom of the figure. The sample size was 20 (*n* = 20) at the start for the Sham through T-Z groups; the sample sizes provided in the table reflect reductions during the study because of euthanasia due to aging-related symptoms.

At 6 months of age, the rats were randomly assigned to one of the following body weight-matched groups: baseline (B) group (*n* = 6), OVX group (*n* = 172), or Sham group (*n* = 32). Rats in the OVX and Sham groups underwent bilateral ovariectomy or sham ovariectomy, respectively, while rats in the B group were sacrificed just prior to surgery by exsanguination from the abdominal aorta while under general anesthesia. At 2 months postoperatively (*t* = 0 months), the OVX and Sham rats were randomly assigned to either a main treatment group (*n* = 20 per group) or a satellite group. The groups were matched by proximal tibial BMD and body weight as closely as possible to minimize the differences between them (Fig. 1). Medications were then administered over 8 months divided into two 4-month treatment periods. The effects of sequential ZOL–TPTD and TPTD–ZOL treatments were assessed by treating rats with ZOL during the first 4-month treatment period followed by TPTD during the second 4-month treatment period, or vice versa. The ZOL–TPTD treatment set comprised three groups: the ZOL to TPTD sequential treatment group (Z-T), ZOL to vehicle sequential treatment group (Z-V), and vehicle to TPTD sequential treatment group (V-T). The TPTD–ZOL treatment set also comprised three groups: the TPTD to ZOL sequential treatment group (T-Z), TPTD to vehicle sequential treatment group (T-V), and vehicle to ZOL sequential treatment group (V-Z). Untreated (V-V) OVX and Sham rats were used as controls for both groups (Fig. 1). Satellite groups that were sacrificed before the end of the treatment were set as follows: S0, sham-operated rats sacrificed just prior to the start of treatment (*n* = 6; *t* = 0 months); V0, OVX rats sacrificed just prior to the start of treatment (*n* = 8; *t* = 0 months); S4, sham-operated rats sacrificed after 4 months of treatment with vehicle (*n* = 6; *t* = 4 months); V4, OVX rats sacrificed after 4 months of treatment with vehicle (*n* = 8; *t* = 4 months); Z4, OVX rats sacrificed after 4 months of treatment with ZOL (*n* = 8; *t* = 4 months); T4, OVX operated rats sacrificed after 4 months of treatment with TPTD (*n* = 8; *t* = 4 months). The experimental groups are shown in Fig. 1.

### 2.2 Experimental treatments and procedures

The S0 and V0 groups were sacrificed just prior to the start of treatment (*t* = 0 months). The other groups were then treated for 4 months (first treatment period) with vehicle (saline 1 mL/kg, single intravenous (IV) injection and saline 0.5 mL/kg/dose, 3 times/week subcutaneous (SC) injection), ZOL (zoledronic acid 100 μg/kg, single IV and saline 0.5 mL/kg/dose, 3 times/week SC), or TPTD (saline 1 mL/kg, single IV and teriparatide 6.0 μg/kg/dose, 3 times/week SC). A total of 100 μg/kg of zoledronic acid (Zometa; Novartis Pharma K.K., Tokyo, Japan) and 6.0 μg/kg of teriparatide (Asahi Kasei Pharma Corp., Tokyo, Japan) were prepared in saline for each injection. At the end of the first treatment period (*t* = 4 months), the S4, V4, Z4, and T4 groups were sacrificed and the main treatment groups were switched to their second period treatments (Fig. 1). After 4 more months (end of the second treatment period; *t* = 8 months), all remaining groups (*n* = 14–20/group) were sacrificed. Although all of the main treatment groups started with 20 animals at the beginning of the first treatment period, the group sizes reduced over time due to euthanasia of some rats for age-related symptoms before the end of the study (Fig. 1). The fourth and fifth lumbar vertebrae (L4 and L5) and the right femur of each rat were harvested at the end of the second treatment period for further analysis.

### 2.3 Dosage and administration

The typical clinical dosage and administration protocol for ZOL is 5 mg administered by intravenous infusion once yearly. Assuming that the average human body weight is 50 kg, the per body weight dosage is 100 μg/kg. One previous study reported that OVX rats given a single intravenous administration at this dose had BMD and bone strength values at 8 months post-dose that were equivalent to or greater than those of sham-operated animals (Gasser et al., 2008). Thus, we administered ZOL to the OVX rats in the current study at a dose of 100 μg/kg. In extrapolating a once-yearly treatment from humans to rats, consideration must be given to the rate of bone turnover in osteoporotic women and OVX rats. Because bone turnover in OVX rats (Takakura et al., 2016, Ma et al., 1995) is at least three times faster (Takakura et al., 2016) than in women with postmenopausal osteoporosis (Miki et al., 2004, Chavassieux et al., 1997), we selected a dosing frequency of once every 4 months for the treatment of the OVX rats in the present study.

TPTD is available in both a daily and a once-weekly formulation. The approved dosage and administration protocol for the once-weekly formulation of TPTD is 56.5 μg/kg by subcutaneous injection once per week. In past studies, we assessed dosing frequencies and doses of TPTD in rats. The area under the blood concentration-time curve (AUC) in rats given 5.6 μg/kg of TPTD by subcutaneous injection three times weekly was 79.992 ng⋅min/ml (Sugie-Oya et al., 2016), which was equivalent to the AUC of 55.5–74.1 ng⋅min/ml found in human subjects given 56.5 μg/kg of TPTD once weekly (Sugimoto et al., 2014). Under these treatment conditions, the bone strength of OVX rats after 4 months of treatment has been shown to be similar to that of sham-operated animals (Sugie-Oya et al., 2016). Thus, in the present study, the dosage and administration protocol selected for TPTD was 6.0 μg/kg administered subcutaneously three times weekly.

### 2.4 Measurement of metabolic markers of bone turnover in urine and serum samples

The treatment groups were fasted for > 6 h before blood and urine collection at the following experimental time points: 0, 1, 2, 4, 5, 6, and 8 months. Overnight urine samples were obtained the night before drug administration. Serum samples were obtained by centrifugation of blood samples collected from the subclavian vein before drug administration on the morning of the administration day. The urine and serum samples were aliquoted and stored at − 80 °C until analysis. The urinary concentration of type I collagen cross-linked C-telopeptide (CTX), a marker of bone resorption, was measured using RatLaps EIA (Immunodiagnostic Systems Ltd., Boldon, UK) and corrected for the urinary creatinine concentration. The urinary creatinine concentration was determined using L-type Wako CRE·M (Wako Pure Chemical Industries, Ltd., Osaka, Japan). The serum concentration of osteocalcin (OC), a marker of bone formation, was determined using an osteocalcin rat ELISA (GE Healthcare Japan Corp., Tokyo, Japan). All assays were performed in accordance with the manufacturers' instructions.

### 2.5 Skeletal analyses

#### 2.5.1 Preparation of bone samples

The L4 vertebrae and right femurs were harvested at the end of the treatment period. Prior to measurement, the bone samples were soaked at room temperature and cleaned to remove adherent soft tissues. The vertebral arch and the transverse and spinous processes were removed from each L4 vertebrae. The L4 vertebral body was then processed using a diamond bandsaw (BS-3000; Exakt, Norderstedt, Germany) to obtain a central cylindrical specimen with parallel ends and a height of 3.8 ± 0.1 mm for further testing. This procedure removed both the cartilaginous growth plate and primary spongiosa (Mosekilde et al., 1993).

The L5 vertebrae were harvested at the end of the treatment period and dissected free of soft tissues, fixed in 70% ethanol, stained with Villanueva bone stain, dehydrated in a graded ethanol series, defatted in acetone, and embedded in methyl methacrylate (Wako Pure Chemical Industries, Ltd.). Thin sections (5 μm) were prepared from sagittal sections of L5.

#### 2.5.2 Measurement of BMD

Changes in tibial BMD were monitored in vivo with the rats under intraperitoneal pentobarbital anesthesia at − 2, 0, 1, 2, 4, 5, 6, and 8 months using dual-energy X-ray absorptiometry (DXA) (DCS-600EX-3R; Hitachi Ltd., Tokyo, Japan), which had a coefficient of variation < 1%. The rats were anesthetized and placed in a prone position on the DXA scanning table, and their left tibias were scanned at a pitch of 1 mm and speed of 25 mm/min. Areal BMD (mg/cm^2^) was calculated from the bone mineral content (mg) and bone area (cm^2^). The tibia was then divided into three equal-length regions of interest, and the BMD of the proximal region was obtained.

The BMDs of the L4 vertebral cylinders and right femurs were measured using DXA (DCS-600EX-3R; Hitachi Ltd.). The specimens were placed on the scanning table ventral side up and scanned at a pitch of 1 mm and speed of 25 mm/min. The BMD (mg/cm^2^) was calculated from the bone mineral content (mg) and bone area (cm^2^). The femur was divided into three equal-length regions of interest for analysis (proximal, diaphysis, and distal), and the BMD of each region was obtained.

#### 2.5.3 Bone histomorphometry

For bone histomorphometric analysis, the treatment groups underwent in vivo bone staining by subcutaneous injection of 10 mg/kg calcein (Dojindo Laboratories, Kumamoto, Japan) and 20 mg/kg tetracycline (Sigma-Aldrich Corp., St. Louis, MO, USA) at 13 and 3 days, respectively, before sacrifice.

Bone histomorphometric parameters related to bone mass, resorption, formation, and turnover were measured using an image analysis system (Histometry RT Camera; System Supply Co., Ltd., Nagano, Japan). Histomorphometric measurements were performed using cancellous bone tissue from the secondary spongiosa region of the L5 vertebra, defined as the interior of the lumbar vertebra 1.0 mm from the cranial and caudal growth plates and 0.5 mm from the cortical bone. The following static and dynamic parameters were measured: bone volume (BV/TV), trabecular thickness (Tb.Th), trabecular number (Tb.N), osteoid surface (OS/BS), osteoblast surface (Ob.S/BS), mineralizing surface (MS/BS, based on double plus half single label), single label surface (sLS/BS), double label surface (dLS/BS), bone formation rate per bone volume (BFR/BV), eroded surface (ES/BS), and osteoclast surface (Oc.S/BS) (Parfitt et al., 1987). Tb.N, OS/BS, sLS/BS, dLS/BS, BFR/BV are reported only in the supplemental data (Suppl. Table 3).

#### 2.5.4 Mechanical properties of bone

Compression testing of the vertebral body was performed as previously described (Mosekilde et al., 1993). The cylindrical specimens from the L4 vertebrae were placed cranial side up on a lower platen and compressed with a 4-mm-diameter upper platen using a materials testing machine (EZ-L-1kN; Shimadzu Corp., Tokyo, Japan) at a constant speed of 2 mm/min. The load and displacement curves were recorded, and the following extrinsic parameters were calculated by the testing machine's software (TRAPEZIUM X; Shimadzu Corp.): maximum load (N), stiffness (N/mm), and breaking energy (hereafter referred to as energy; N⋅mm).

The femoral diaphysis was subjected to a three-point bending test as previously described (Molster, 1986) using the materials testing machine at a constant speed of 10 mm/min. The maximum load (N) was recorded. After the three-point bending test, mechanical testing of the proximal femur was performed as previously described (Sogaard et al., 1994) using the materials testing machine. The shaft of the proximal femur was embedded vertically in dental resin. The specimen was placed on a lower platen with the proximal part facing up and compressed on a 4-mm-diameter upper platen at a constant speed of 2 mm/min. The maximum load (N) was recorded.

#### 2.5.5 Cortical geometry of the femoral diaphysis

A cone-beam X-ray micro-CT system (ScanXmate-RB090SS150; Comscantecno, Kanagawa, Japan) was used to obtain CT images of the femoral diaphysis of isolated bone samples using the following settings: tube voltage, 70 kV; tube current, 0.1 mA; and an isotropic voxel size of 12.53 μm. Three-dimensional images were reconstructed and analyzed using TRI/3D–BON software (RATOC System Engineering, Tokyo, Japan). A region (height, 1.0 mm) on the plane perpendicular to the shaft at a point halfway along the length of the femur was analyzed. The cortical bone structure was determined in the femoral diaphysis, and the following parameters were measured: cortical bone thickness (Ct.Th, μm), external length (Ex.L, μm), internal length (In.L, μm), and three dimensional moment of inertia (moment, mm^5^) around the mediolateral axis.

#### 2.5.6 Statistical analysis

Data are presented as means ± standard deviation (S.D.).

To assess the effects of the OVX procedure, differences between the OVX and sham groups were assessed using Student's *t-*tests for all data at any time point.

To assess the efficacy of each treatment, each treatment group was compared with the OVX group. Data were subjected to one-way analysis of variance, and between-group differences were analyzed using a Dunnett-type test for all parameters at each time point.

To assess in detail the effects of sequential treatments, the following parameters were analyzed: proximal tibial BMD at months 5, 6, and 8; serum osteocalcin at months 5, 6, and 8; urinary CTX at months 5, 6, and 8; bone strength of the lumbar vertebral body and femur; and morphometric measurements of the resected lumbar vertebral body. The effects of TPTD after ZOL treatment were assessed by a *t*-test comparing the Z-T group with the Z-V group, and the effects of ZOL after TPTD treatment were assessed by a *t*-test comparing the T-Z group with the T-V group.

Parametric tests were used for the analyses on the premise that all the data had equal variance, as we have shown in previous studies. All statistical analyses were performed with SAS software (SAS Institute Inc., Cary, NC, USA). *P* values < 0.05 were considered significant.

## 3 Results

### 3.1 Sequential ZOL to TPTD treatment

#### 3.1.1 BMD

**Fig. 2:**
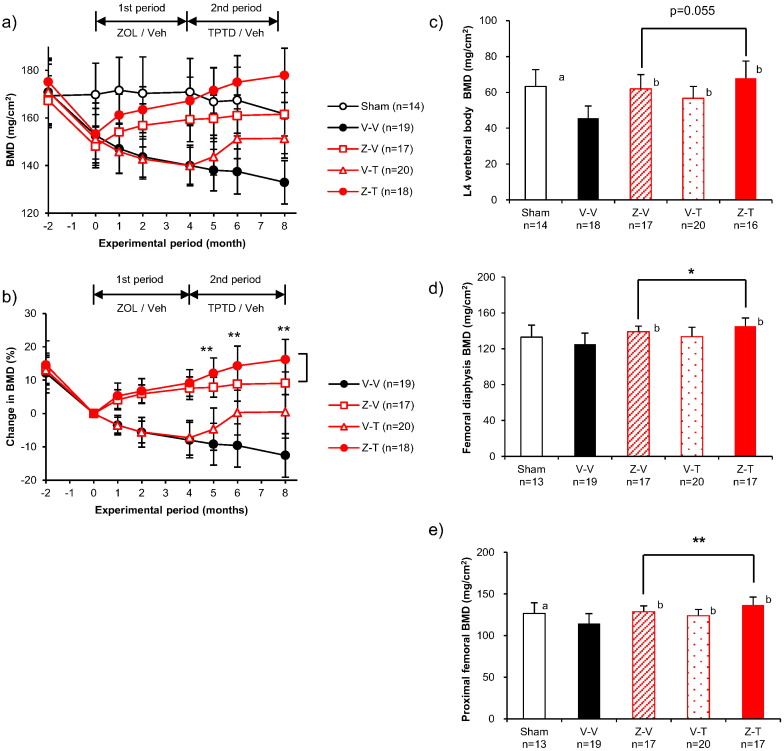
Bone mineral density of the ZOL to TPTD set. (a) BMD at the proximal tibia over time measured by in vivo DXA. (b) Percent changes in BMD at the proximal tibia over time with the percent change at month 0 set as 0%. The sham-operated group has been omitted from the graphs showing percent changes in BMD. BMD at the (c) 4th lumbar vertebral body, (d) femoral diaphysis, and (e) proximal femur at the study conclusion (month 8). See Table 1 for the satellite group results from before the OVX operation (group B), immediately before the first treatment period (groups S0 and V0), and immediately before the second treatment period (groups S4, V4, Z4, and T4). *: *p* < 0.05, **: *p* < 0.01, significant difference between the Z-T group and Z-V group during each experimental period (Student's *t*-test). a: *p* < 0.05, significant difference between Sham vs V-V (Student's *t*-test). b: *p* < 0.05, significant difference vs V-V (Dunnett-test). Data are presented as means ± S.D.

The BMD at the proximal tibia increased over the first 4 months of treatment (the ZOL period) in the ZOL-treated groups (Z-T, Z-V; Fig. 2b). In the second treatment period, BMD increased from the month 4 value at months 5, 6, and 8 (2.7%, 4.7%, and 6.4%, respectively) in the Z-T group, but did not increase after month 4 in the Z-V group. BMD was significantly higher in the Z-T group than in the Z-V group at months 5, 6, and 8 (Fig. 2b).

Analysis of bones resected at 8 months after initiation of treatment revealed several between-group differences. The BMD of the fourth lumbar vertebra (L4) was greater in the Z-T group than in the Z-V group, although the difference was not statistically significant (10%; *p* = 0.055; Fig. 2c, Table 1). The BMD of the femoral diaphysis was significantly greater in the Z-T group than in the Z-V group (4%; *p* < 0.05; Fig. 2d, Table 1). The BMD of the proximal femur was significantly greater in the *Z*-T group than in the Z-V group (6%; *p* < 0.01; Fig. 2e, Table 1).

**Table 1:** Ex vivo BMD and bone strength parameters at the 4th lumbar vertebral body, femoral diaphysis, and proximal femur. B: baseline, non-operated group, euthanized at the time of the OVX operation; S0: satellite group, sham-operated, euthanized at the start of the first treatment period; V0: satellite group, ovariectomized, euthanized at the start of the first treatment period; S4: satellite group, sham-operated, euthanized at the end of the first treatment period; V4: satellite group, ovariectomized, euthanized at the end of the first treatment period; Z4: satellite group, ovariectomized, euthanized at the end of the first treatment period, ZOL-treated; T4: satellite group, ovariectomized, euthanized at the end of first treatment period, TPTD-treated. a: *p* < 0.05, significant difference between Sham groups (S0, S4 or Sham) and OVX groups (V0, V4 or V-V) at each time point (Student's *t*-test). b: *p* < 0.05, significant difference vs V4 (Dunnett-test). c: *p* < 0.05, significant difference vs V-V (Dunnett-test). d: *p* < 0.05, significant difference between the Z-T group and Z-V group. e: *p* < 0.05, significant difference between the T-Z group and T-V group. (S.D. = standard deviation).

		B	S0	V0	S4	V4	Z4	T4	Sham	V-V	Z-V	V-T	Z-T	T-V	V-Z	T-Z
*4th Lumbar vertebral body*																
BMD (mg/cm^2^)	Mean	75.5	67.4^a^	59.6	60.2^a^	52.1	60.4	60.6	63.3^a^	45.5	61.9^c^	56.7^c^	68.0^c^	46.6	52.9^c^	62.9^c,e^
	S.D.	4.3	6.3	4.1	3.7	4.0	5.0	10.7	9.4	6.9	8.0	6.6	9.5	5.5	5.4	9.2
	n	4	6	7	5	7	8	8	14	18	17	20	16	17	18	17
Maximum load (N)	Mean	448.1	352.6	316.2	283.5^a^	228.2	315.7	354.0^b^	280.4^a^	205.7	316.2^c^	293.6^c^	394.9^c,d^	207.8	270.7^c^	355.2^c,e^
	S.D.	33.3	79.0	49.2	51.0	34.4	47.1	115.1	71.1	68.4	56.0	45.0	82.7	41.3	56.0	71.4
	n	4	5	7	5	7	8	8	14	18	16	20	16	17	18	16
Stiffness (N/mm)	Mean	2284.8	2188.9^a^	1996.6	1950.1	1686.2	1965.4	2078.2^b^	1905.5^a^	1539.6	2031.9^**c**^	1905.8^c^	2219.7^c,d^	1636.4	1853.2^c^	2090.5^c,e^
	S.D.	119.0	123.7	84.8	211.9	232.3	182.8	393.6	225.2	326.2	187.7	208.0	251.0	239.4	234.3	164.9
	n	4	5	7	5	7	8	8	14	18	16	20	16	17	18	16
Energy (N/mm)	Mean	54.6	38.2	33.5	27.5	22.3	33.3	39.6^b^	28.2^a^	18.7	33.3^c^	31.2^c^	44.7^c,d^	18.3	26.9^c^	39.7^c,e^
	S.D.	6.7	12.5	14.2	5.1	4.3	6.1	18.7	13.2	9.2	8.0	7.4	13.0	4.5	7.5	11.9
	n	4	5	7	5	7	8	8	14	18	16	20	16	17	18	16

*Femoral diaphysis*																
BMD (mg/cm^2^)	Mean	124.2	128.3	126.7	135.9^a^	125.0	135.3^b^	139.9^b^	133.0	125.3	139.2^c^	133.6	145.3^c,d^	125.8	131.1	138.4^c,e^
	S.D.	6.8	4.8	8.8	5.9	7.2	8.1	10.5	13.5	12.2	6.1	10.3	9.1	10.2	8.5	11.5
	n	6	6	8	6	8	8	8	13	19	17	20	17	18	18	18
Maximum load (N)	Mean	164.2	175.9	183.0	189.9	195.6	203.6	189.9	208.6	192.1	197.1	205.7	232.2^c,d^	181.7	197.1	216.6^e^
	S.D.	24.7	18.6	22.6	13.8	25.0	13.9	40.4	32.6	30.0	29.2	31.8	41.0	51.7	24.6	41.3
	n	6	6	8	6	8	8	8	13	19	17	20	17	18	18	18
Stiffness (N/mm)	Mean	449.5	437.7	478.0	492.5	503.1	472.3	518.3	513.3	456.1	462.2	486.3	552.5^c,d^	473.3	472.7	536.7^c^
	S.D.	40.9	84.0	62.8	44.4	60.5	63.0	49.1	77.4	93.0	77.1	70.3	73.7	102.3	68.5	100.8
	n	6	6	8	6	8	8	8	13	19	17	20	17	18	18	18
Energy (N/mm)	Mean	58.8	56.2	61.2	58.0	85.0	69.7	65.4	77.7	71.4	66.5	74.5	87.7^d^	62.7	70.6	73.0
	S.D.	19.9	14.1	14.0	11.3	33.2	9.4	28.5	20.3	13.9	14.1	22.4	35.1	36.3	12.5	21.1
	n	6	6	8	6	8	8	8	13	19	17	20	17	18	18	18

*Proximal femur*																
BMD (mg/cm^2^)	Mean	128.9	133.6^a^	122.0	131.9^a^	116.9	126.8^b^	134.2^b^	126.4^a^	114.5	128.7^c^	124.0^c^	136.8^c,d^	115.9	119.8	130.8^c,e^
	S.D.	7.0	5.8	8.7	5.6	7.2	6.6	9.7	13.1	11.9	7.0	7.4	9.4	10.3	8.9	11.8
	n	6	6	8	6	8	8	8	13	19	17	20	17	18	18	18
Maximum load (N)	Mean	109.7	124.0	122.6	114.3^a^	91.8	101.4	113.4^b^	119.0^a^	102.4	107.6	114.8	133.8^c,d^	105.9	108.5	119.2^c,e^
	S.D.	27.4	13.7	21.7	15.4	15.0	8.1	8.4	17.8	14.7	13.9	19.5	18.0	15.8	23.0	20.8
	n	6	6	8	6	8	8	8	13	19	17	20	17	18	18	18
Stiffness (N/mm)	Mean	403.3	466.6	472.8	414.4^a^	321.8	391.6^b^	386.8^b^	499.3^a^	416.7	424.9	425.2	516.7^c,d^	434.3	389.7	467.3
	S.D.	83.3	44.6	50.6	55.5	46.3	37.0	64.2	74.8	83.3	69.9	76.2	77.1	73.9	99.2	76.3
	n	6	6	8	6	8	8	8	13	19	17	20	17	18	18	18
Energy (N/mm)	Mean	24.9	23.3	21.4	18.4	16.9	17.5	20.6	19.7	17.0	17.6	20.7	22.4^c,d^	17.8	20.2	19.4
	S.D.	7.6	7.2	6.2	3.0	5.6	4.7	3.6	4.1	6.2	5.3	7.5	4.4	4.9	6.4	4.4
	n	6	6	8	6	8	8	8	13	19	17	20	17	18	18	18

#### 3.1.2 Mechanical properties of bone

**Fig. 3:**
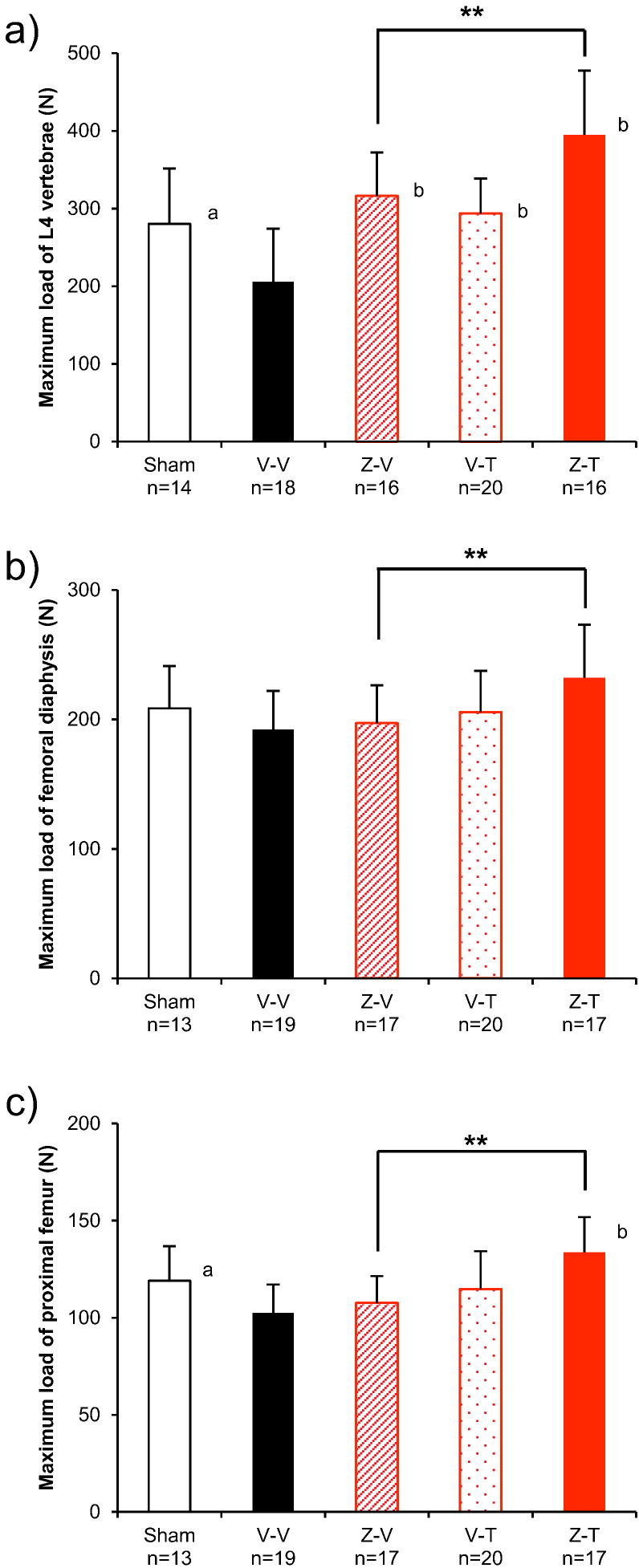
Mechanical properties of the bones resected at the study conclusion from the ZOL to TPTD set. (a) maximum compression test loads withstood by the L4 vertebral body, (b) maximum three-point bending test loads withstood by the femoral diaphysis, and (c) maximum mechanical test loads withstood by the proximal femur. See Table 1 for the satellite group results before the OVX operation (group B), immediately before the first treatment period (groups S0 and V0), and immediately before the second treatment period (groups S4, V4, Z4, and T4).**: *p* < 0.01, significant difference between the Z-T group and Z-V group (Student's *t*-test). a: *p* < 0.05, significant difference between Sham vs V-V (Student's *t*-test). b: *p* < 0.05, significant difference vs V-V (Dunnett-test). Data are presented as means ± S.D.

The maximum load withstood by L4 at 8 months after treatment initiation was significantly higher in the Z-T group than in the Z-V group (25%; *p* < 0.01; Fig. 3a, Table 1). The maximum load withstood by the femoral diaphysis at 8 months after treatment initiation was also significantly higher in the Z-T group than in the Z-V group (18%; *p* < 0.01; Fig. 3b, Table 1). The maximum load withstood by the proximal femur at 8 months after treatment initiation was significantly higher in the Z-T than in the Z-V group (24%; *p* < 0.01; Fig. 3c, Table 1). Similarly, the stiffness and energy of L4, the femoral diaphysis, and the proximal femur were also significantly higher in the Z-T group than in the Z-V group (Table 1).

#### 3.1.3 Markers of bone turnover

**Fig. 4:**
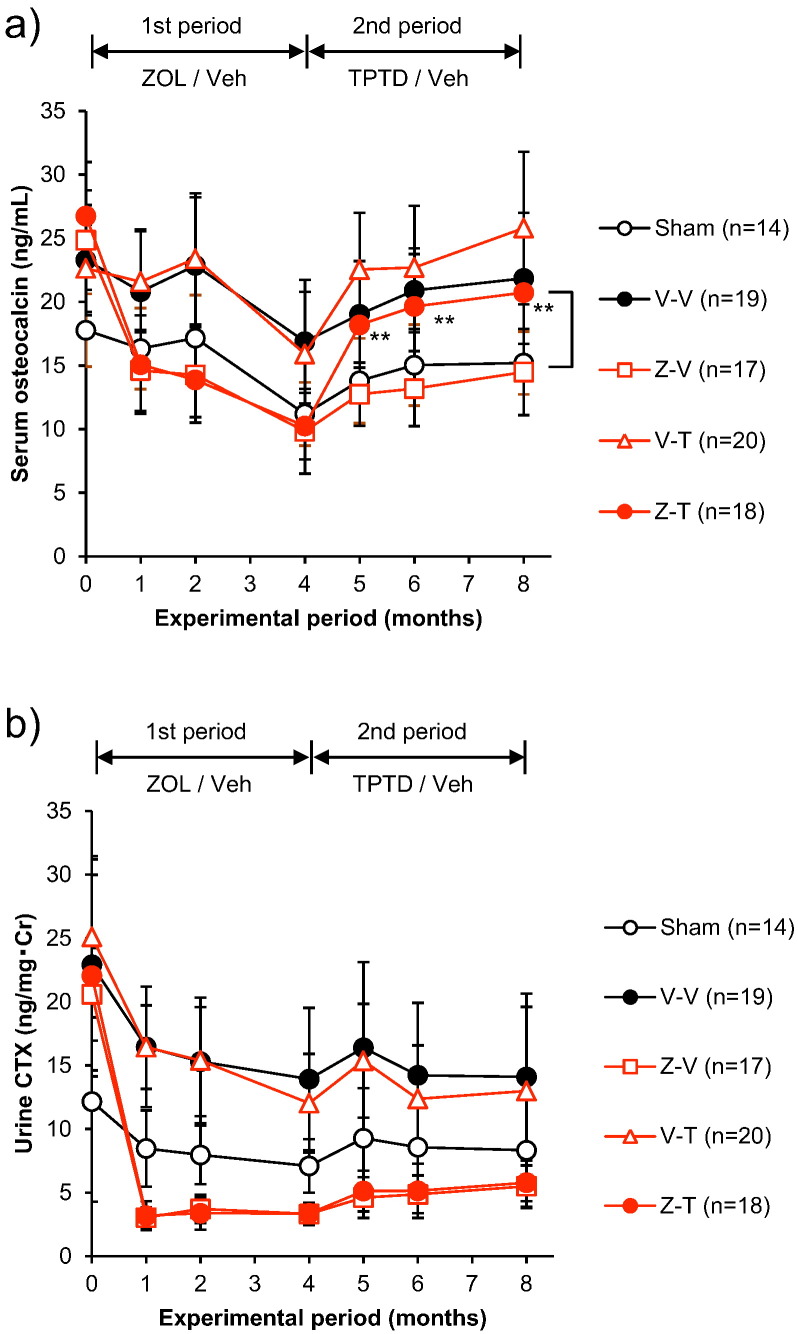
Change in markers of bone metabolism for the ZOL to TPTD set. (a) serum osteocalcin concentration, (b) urinary CTX concentration corrected for creatinine. **: *p* < 0.01, significant difference between the Z-T group and Z-V group during each experimental period (Student's *t*-test). Data are presented as means ± S.D.

The groups given ZOL during the first treatment period (Z-T, Z-V) had significantly lower serum osteocalcin concentrations than the V-V group at months 1, 2, and 4 (Suppl. Table 2). During the second treatment period, the serum osteocalcin concentration increased in the Z-T group to become significantly higher than that of the Z-V group and reach the level of the V-V group at months 5, 6, and 8. In the Z-V group, the serum osteocalcin concentration remained lower than in the V-V group at months 5, 6, and 8 (Fig. 4a).

In the groups given ZOL during the first treatment period (Z-T, Z-V), the urinary CTX concentration was significantly lower than in the V-V group at months 1, 2, and 4 (Suppl. Table 2). Even in the Z-T group, urinary CTX was equivalent to that of the *Z*-V group and remained lower than that of the V-V group at months 5, 6, and 8 (Fig. 4b).

#### 3.1.4 Bone histomorphometry

**Fig. 5:**
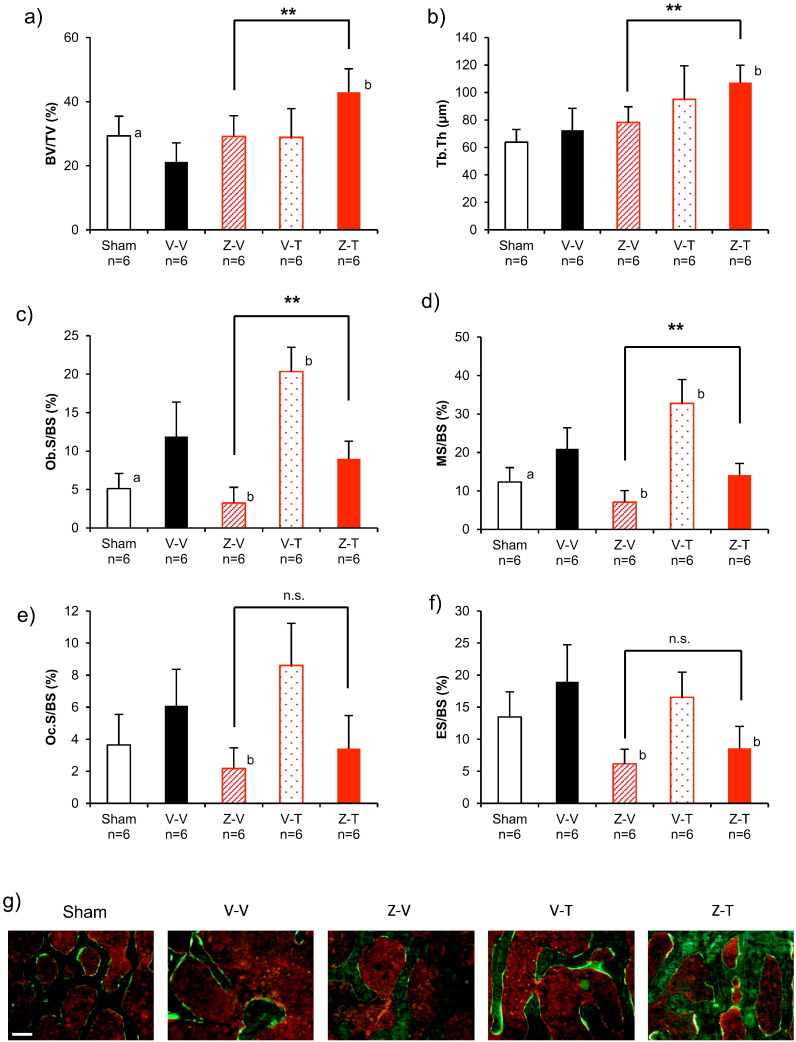
Results of histomorphometry of the L5 vertebral body for the ZOL to TPTD set. (a) bone volume (BV/TV), (b) trabecular thickness (Tb.Th), (c) osteoblast surface (Ob.S/BS), (d) mineralizing surface (MS/BS), (e) osteoclast surface (Oc.S/BS), (f) eroded surface (ES/BS), (g) representative fluorescent images of each group.**: *p* < 0.01, significant difference between the Z-T group and Z-V group (Student's *t*-test). a: *p* < 0.05, significant difference between Sham vs V-V (Student's *t*-test), b: *p* < 0.05, significant difference vs V-V (Dunnett-test). Data are presented as means ± S.D. Scale bar = 200 μm

Histomorphometry of L5 showed that both BV/TV and Tb.Th were greater in the Z-T group than in the Z-V group (Fig. 5a, b, Suppl. Table 3). In addition, the bone formation parameters Ob.S/BS and MS/BS were greater in the Z-T group than in the Z-V group (Fig. 5c, d, Suppl. Table 3). There were no between-group differences in the resorption parameters ES/BS and Oc.S/BS (Fig. 5e, f, Suppl. Table 3).

#### 3.1.5 Cortical geometry of the femoral diaphysis

The moment of the femoral diaphysis at 8 months after treatment initiation was significantly higher in the Z-T group than in the Z-V group (11%; *p* < 0.05; Table 2). No differences were noted in Ct.Th, In.L, or Ex.L.

**Table 2:** Cortical geometry of the femoral diaphysis. a: *p* < 0.05, significant difference between Sham and V-V groups (Student's *t*-test). b: *p* < 0.05, significant difference vs V-V (Dunnett-test). c: *p* < 0.05, significant difference between the Z-T group and Z-V group. d: *p* < 0.05, significant difference between the T-Z group and T-V group. (S.D. = standard deviation).

		Sham	V-V	Z-V	V-T	*Z*-T	T-V	V-Z	T-Z
*Femoral diaphysis*									
Cortical thickness (μm)	Mean	720.6^a^	643.3	789.2^b^	733.1^b^	801.7^b^	643.2	712.2^b^	777.0^**b,d**^
	S.D.	72.2	88.6	41.0	60.8	68.3	94.2	82.9	76.1
	n	13	19	17	20	17	17	18	18
Moment (mm^5^)	Mean	8.0	8.4	8.5	8.6	9.4^c^	8.4	8.7	8.3
	S.D.	1.5	1.3	1.1	1.5	1.3	1.2	1.2	1.4
	n	13	19	17	20	17	17	18	18
Internal length (mm)	Mean	7.52^a^	8.58	7.15^b^	7.66^b^	7.39^b^	8.73	7.97	7.17^b,d^
	S.D.	0.66	1.10	0.59	0.55	0.53	1.21	0.89	0.71
	n	13	19	17	20	17	17	18	18
External length (mm)	Mean	12.23	12.52	12.31	12.39	12.62	12.62	12.58	12.15^d^
	S.D.	0.65	0.65	0.43	0.57	0.51	0.60	0.54	0.57
	n	13	19	17	20	17	17	18	18

### 3.2 Sequential TPTD to ZOL treatment

#### 3.2.1 BMD

**Fig. 6:**
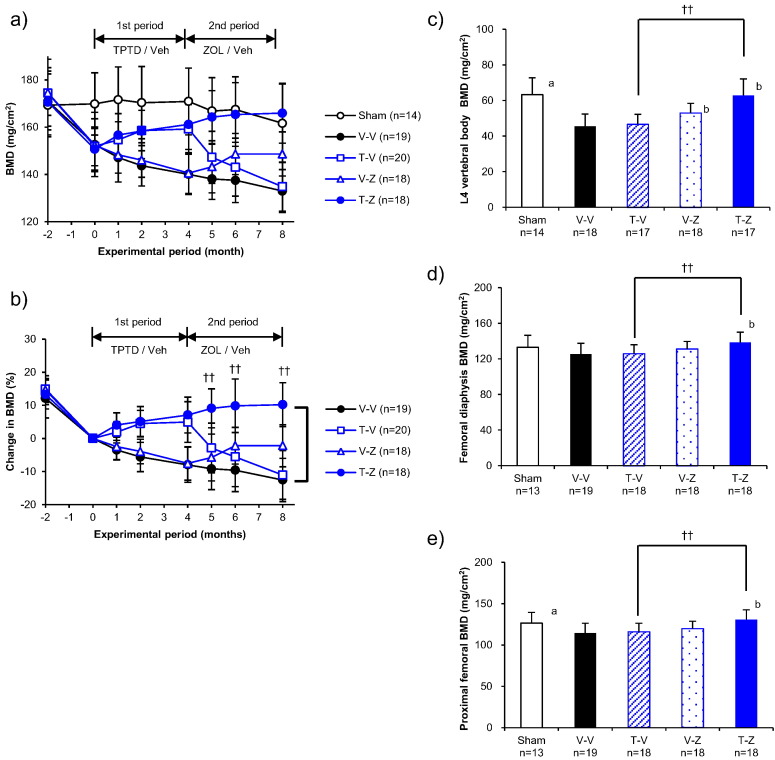
Bone mineral density of the TPTD to ZOL set. (a) BMD values at the proximal tibia over time measured by in vivo DXA. (b) Percent change in BMD at the proximal tibia over time, with the percent change at month 0 set as 0%. The sham-operated group has been omitted from the graphs showing percent changes in BMD. BMD at the (c) L4 vertebral body, (d) femoral diaphysis, and (e) proximal femur at the study conclusion (month 8). See Table 1 for the satellite group results from before the OVX operation (group B), immediately before the first treatment period (groups S0 and V0), and immediately before the second treatment period (groups S4, V4, Z4, and T4). ^††^: *p* < 0.01, significant difference between the T-Z group and T-V group during each experimental period (Student's *t*-test). a: *p* < 0.05, significant difference between Sham vs V-V (Student's *t*-test). b: *p* < 0.05, significant difference vs V-V (Dunnett-test). Data are presented as means ± S.D.

In vivo measurements made using DXA revealed between-group differences in progressive changes to BMD. In the groups given TPTD during the first treatment period (T-Z, T-V), BMD at the proximal tibia increased over 4 months (Fig. 6b). However, BMD at the proximal tibia decreased between months 4 and 8 in the T-V group, while in the T-Z group BMD increased from the month 4 value at months 5, 6, and 8 (1.9%, 2.6%, and 2.9%, respectively). Moreover, BMD was significantly greater in the T-Z group than in the T-V group at months 5, 6, and 8 (Fig. 6b).

Ex vivo BMD measurements made using bones resected at month 8 also revealed significant between-group differences. The T-Z group had significantly greater BMD than the T-V group at L4 (35%; *p* < 0.01; Fig. 6c, Table 1), the femoral diaphysis (10%; *p* < 0.01; Fig. 6d, Table 1), and the proximal femur (13%; *p* < 0.01; Fig. 6e, Table 1).

#### 3.2.2 Mechanical properties of bone

**Fig. 7:**
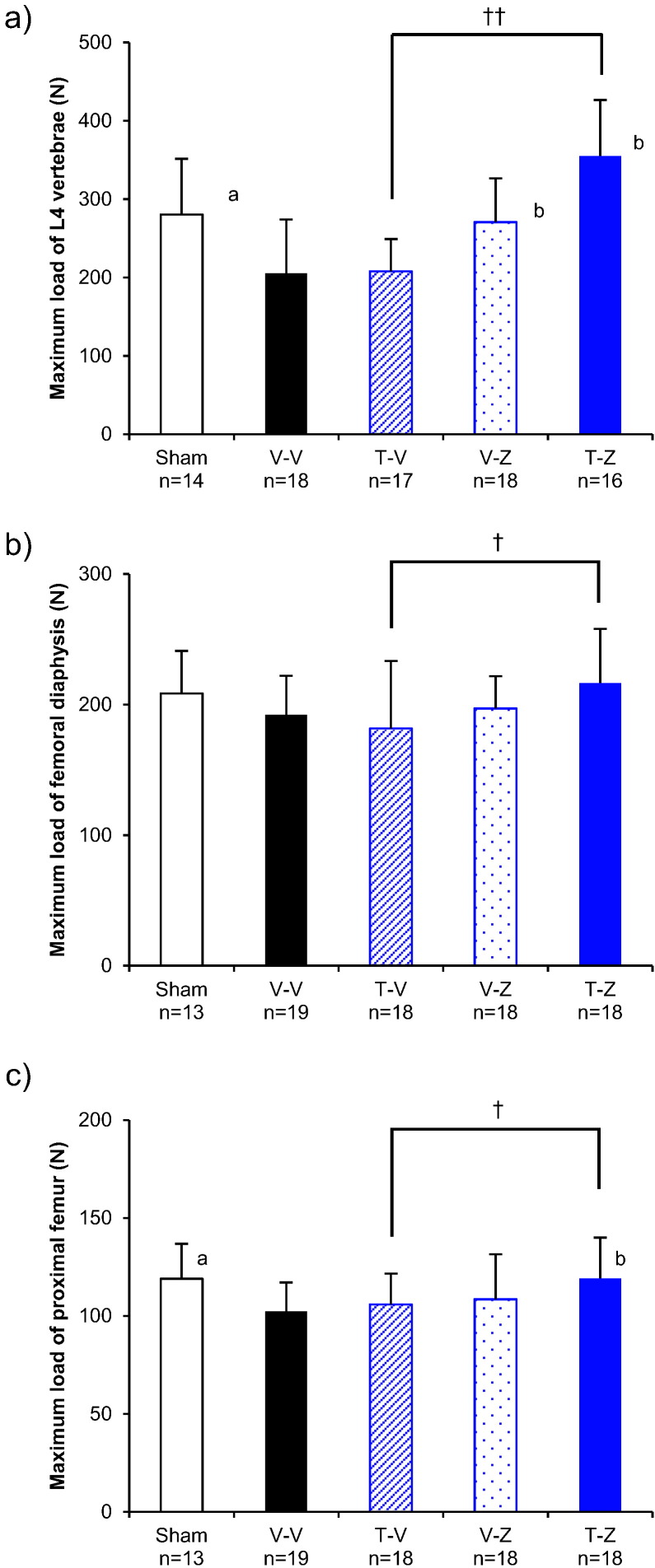
Mechanical properties of the bones resected at the study conclusion from the TPTD to ZOL set. (a) maximum compression test loads withstood by the L4 vertebral body, (b) maximum three-point bending test loads withstood by the femoral diaphysis, and (c) maximum mechanical testing loads withstood by the proximal femur. See Table 1 for the satellite groups results before the OVX operation (group B), immediately before the first treatment period (groups S0 and V0), and immediately before the second treatment period (groups S4, V4, Z4, and T4). ^†^: *p* < 0.05, ^††^: *p* < 0.01, significant difference between the T-Z group and T-V group during each experimental period (Student's *t*-test). a: *p* < 0.05, significant difference between Sham vs V—V (Student's *t*-test). b: *p* < 0.05, significant difference vs V-V (Dunnett-test). Data are presented as means ± S.D.

The maximum load withstood by L4 at 8 months after treatment initiation was significantly greater in the T-Z group than in the T-V group (71%; *p* < 0.01; Fig. 7a, Table 1). Similar results were obtained with respect to stiffness and energy (Table 1).

The maximum load withstood by the femoral diaphysis was significantly greater in the T-Z group than in the T-V group (19%; *p* < 0.05; Fig. 7b, Table 1). Stiffness also tended to be greater in the T-Z group than in the T-V group, although the difference was not significant (13%; *p* = 0.070). Energy tended to be higher in the T-Z group than in the T-V group, although this difference was also not significant (16%; *p* = 0.304; Table 1).

The maximum load withstood by the proximal femur was significantly greater in the T-Z group than in the T-V group (13%; *p* < 0.05; Fig. 7c, Table 1). No significant differences in stiffness or energy were noted between the T-Z and T-V groups.

#### 3.2.3 Markers of bone turnover

**Fig. 8:**
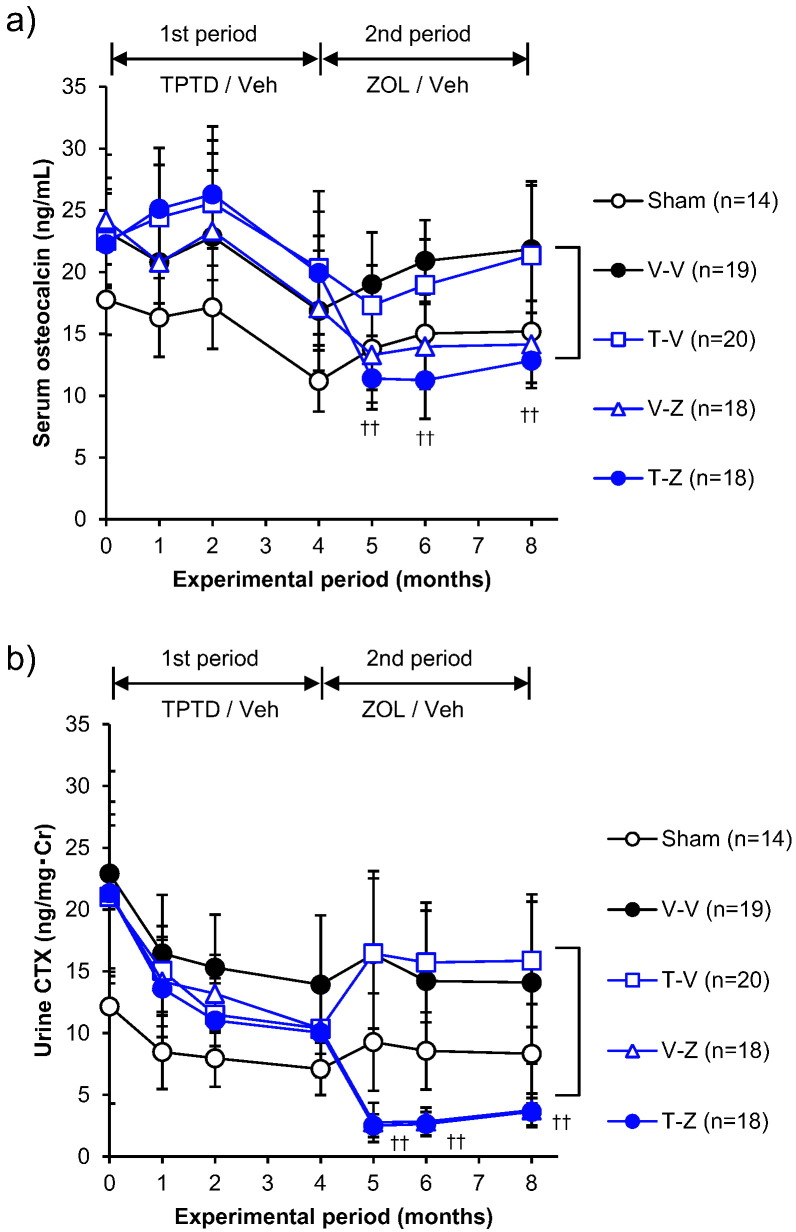
Changes in markers of bone metabolism for the TPTD to ZOL set. (a) serum osteocalcin concentration, (b) urinary CTX concentration corrected for creatinine.^††^: *p* < 0.01, significant difference between the T-Z group and T-V group during each experimental period (Student's *t*-test). Data are presented as means ± S.D.

The groups given TPTD during the first treatment period (T-Z, T-V) had serum osteocalcin concentrations that were significantly higher than that of the OVX group at month 1, and tended to be higher than that of the OVX group at months 2 and 4, although the differences were not significant (Suppl. Table 2). The osteocalcin concentration was significantly lower in the T-Z group than in the T-V group at months 5, 6, and 8. The concentration was equivalent between the T-V group and the OVX control group (V-V) at months 5, 6, and 8 (Fig. 8a).

In the groups given TPTD during the first treatment period (T-Z, T-V), the urinary CTX concentration was significantly lower than in the V-V group at months 2 and 4 (Suppl. Table 2). Urinary CTX was also significantly lower in the T-Z group than in the T-V group at months 5, 6, and 8. In the T-V group, the urinary CTX concentration increased to a level equivalent to that of the V-V group at months 5, 6, and 8 (Fig. 8b).

#### 3.2.4 Bone histomorphometry

**Fig. 9:**
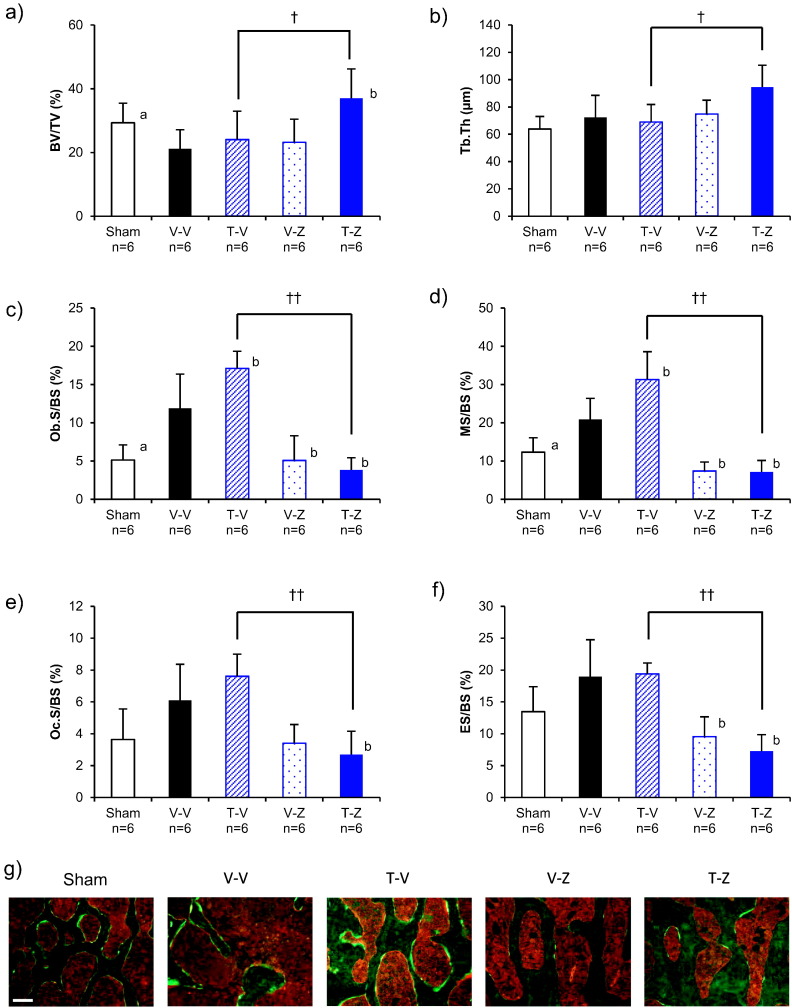
Results of histomorphometry of the L5 vertebral body for the TPTD to ZOL set. (a) bone volume (BV/TV), (b) trabecular thickness (Tb.Th), (c) osteoblast surface (Ob.S/BS), (d) mineralizing surface (MS/BS), (e) osteoclast surface (Oc.S/BS), (f) eroded surface (ES/BS), and (g) representative fluorescent images of each group. ^†^: *p* < 0.05, ^††^: *p* < 0.01, significant difference between the T-Z group and T-V group during each experimental period (Student's *t*-test). a: *p* < 0.05, significant difference between Sham vs V-V (Student's *t*-test). b: *p* < 0.05, significant difference vs V-V (Dunnett-test). Data are presented as means ± S.D. Scale bar = 200 μm

In the T-Z group, BV/TV and Tb.Th were both greater than in the T-V group (Fig. 9a, b, Suppl. Table 3), whereas the bone formation parameters Ob.S/BS and MS/BS, and the bone resorption parameters ES/BS and OcS/BS, were all less than in the T-V group (Fig. 9c, d, e, f, Suppl. Table 3).

#### 3.2.5 Cortical geometry of the femoral diaphysis

The Ct.Th of the femoral diaphysis at 8 months after treatment initiation was significantly higher in the T-Z group than in the T-V group (21%; *p* < 0.01; Table 2). The In.L and Ex.L were both significantly lower in the T-Z group than in the T-V group (− 18%, *p* < 0.01; − 4%, *p* < 0.05, respectively). However, there were no between-group differences in the femoral diaphyseal moment.

## 4 Discussion

In this study, the influences of different sequential treatments with ZOL and TPTD on bone tissues were assessed in OVX rats. Our results showed early increases in BMD at the proximal tibia and increases in BMD and bone strength at the lumbar spine, femoral diaphysis, and proximal femur after the switch from ZOL to TPTD. Meanwhile, the BMD of the proximal tibia decreased in animals that were switched from TPTD treatment to treatment with vehicle; however, the switch from TPTD to ZOL prevented these decreases, as well as decreases in BMD and bone strength at the lumbar spine, femoral diaphysis, and proximal femur. The changes in markers of bone turnover markers and in bone histomorphometric parameters during the treatments were consistent with these findings. Differences in cortical geometry contributed to, but did not fully explain, the between-group differences in bone strength.

We chose to measure changes in BMD over time at the proximal tibia, a region that is rich in trabecular bone and highly responsive to drugs that affect bone metabolism, and a site where in vivo BMD can be measured very precisely using DXA. In addition, ex vivo BMD and bone strength were measured in the resected lumbar vertebrae, femoral diaphysis, and proximal femur; these regions comprise mainly trabecular, mainly cortical, and a mix of trabecular and cortical bone, respectively. Cortical geometry, which influences the bone strength of the femoral diaphysis, was also analyzed. In clinical studies reported to date that assessed the sequential use of a BP followed by TPTD, early increases in BMD at the lumbar spine were attenuated after the switch, but early decreases in BMD at the proximal femur were noted after the switch, revealing site-specific differences in the skeletal response to treatment (Cosman, 2014, Ettinger et al., 2004) (Boonen et al., 2008) (Miller et al., 2008). These differences may be attributable to differences in the effects of the drugs on different types of bone; for example, the lumbar spine, which is rich in trabecular bone, may respond differently than the proximal femur, which consists of both cortical and trabecular bone.

Our results differ from the results of these clinical studies (Ettinger et al., 2004) (Boonen et al., 2008) (Miller et al., 2008), especially regarding the increase in BMD that we observed at the proximal femur and femoral diaphysis in the *Z*-T treatment group (Fig. 2d, e). We found that ZOL followed by TPTD resulted in an immediate increase in BMD at the proximal tibia as soon as 1 month after the switch (Fig. 2b), indicating that the BMD-enhancing effects of TPTD were not inhibited by the previously administered ZOL. Moreover, compared with the group that underwent a washout period following ZOL treatment, the group given TPTD following ZOL treatment showed higher values for the BMD and bone strength parameters of the resected lumbar vertebral body, femoral diaphysis, and proximal femur (Table 1, Fig. 2c, d, e, Fig. 3a, b, c,). In addition to BMD, our data suggest that cortical geometry (moment) may have contributed to the bone strength of the femoral diaphysis (Table 2).

Since BPs reside in the bone matrix and inhibit osteoclast activity, which is coupled to osteoblast activity, the effect of TPTD may be inhibited by residual BP in the bone. Thus, when considering the phenomenon demonstrated in this study, it is necessary to consider whether ZOL administered during the first treatment period remained in the bone during the second treatment period. Residual levels of ZOL have been shown to persist in the bones of rats over a long period of time, with a half-life of between 150 and 200 days (Weiss et al., 2008); this suggests that, in the present study, ZOL administered during the first treatment period remained in the bones and continued to exert its antiresorptive effect during the second treatment period (from month 4 onward) until study completion (month 8). In fact, urinary CTX concentrations (Fig. 4b) and Oc.S/BS and ES/BS values (Fig. 5e, f) in the *Z*-V group showed that the antiresorptive effect of ZOL persisted for the duration of the second treatment period. However, our results showed that the concentration of osteocalcin, a marker of bone formation, began to increase 1 month after starting TPTD treatment in the T-Z group (Fig. 4a). Thus, our findings indicate that, in spite of residual ZOL in the bones, TPTD exerts its expected bone formation effect after the switch from ZOL to TPTD treatment.

In contrast to the between-group differences in serum osteocalcin, no increase was noted in the urinary CTX concentrations of the Z-T group compared with those of the group that underwent a washout period following ZOL treatment (the Z-V group; Fig. 5b). Although this may not seem consistent with the expected effects of TPTD on biomarkers of bone resorption, it is consistent with the available clinical data. In a clinical setting, daily TPTD has been reported to result in increased concentrations of biomarkers of bone resorption (Arlot et al., 2005); however, once-weekly TPTD in humans, which is the dosing frequency that we extrapolated to three times per week in rats for our study, does not (Sugimoto et al., 2014).

In contrast to the results of a previous study that evaluated the sequential use of a BP followed by TPTD (Ettinger et al., 2004) (Boonen et al., 2008) (Miller et al., 2008), our results suggest that the sequential use of ZOL followed by TPTD causes no attenuation of the BMD-enhancing effect of TPTD that is expected in clinical situations. Previous work has shown that sequential administration of RIS followed by TPTD in 9-month-old intact rats (Yao et al., 2007) inhibited the rate of BMD increase at the proximal tibia relative to that of the group that received TPTD following treatment with vehicle. This phenomenon is similar to the attenuation of the increase in lumbar spine BMD that has been reported clinically in association with sequential use of ALN followed by TPTD (Ettinger et al., 2004). It has also been reported that the BMD of the femoral diaphysis was greater in animals treated sequentially with RIS followed by TPTD than in those treated with RIS followed by vehicle (although no analyses of proximal femur BMD were available), which differed from clinical reports that found that the proximal femur BMD did not increase after the switch to TPTD following ALN or RIS treatment (Ettinger et al., 2004, Miller et al., 2008). However, the use of intact rats and a TPTD dosage substantially greater than the typical clinical dosage may have produced results that differed from the changes at the proximal femur seen in patients with postmenopausal osteoporosis after switching to TPTD therapy. The changes in proximal femur BMD seen in our present study likely reflect the efficacy of the treatment protocols in patients with postmenopausal osteoporosis because we used OVX rats as an animal model of postmenopausal osteoporosis and used dosage and administration frequencies for ZOL and TPTD that were similar to those used clinically in humans. Results similar to those seen after sequential use of RIS followed by TPTD (Yao et al., 2007) have also been reported after investigation of the sequential use of ALN followed by a PTH analog in intact rats; however, the dosage of the PTH analog was substantially greater than the dose that would be used clinically in humans (Gasser et al., 2000).

The results that we obtained after sequential use of TPTD followed by ZOL were similar to those of previous studies that used other BP agents. The phenomenon of BMD decreases after termination of TPTD treatment has been reported in clinical and nonclinical studies (Kaufman et al., 2005, Kurland et al., 2004, Sugimoto et al., 2013, Prince et al., 2005, Ejersted et al., 1998, Shahnazari et al., 2011) and prevention of bone loss by BP administration after withdrawal of TPTD has also been reported (Kurland et al., 2004, Sugimoto et al., 2013, Prince et al., 2005, Ejersted et al., 1998, Iwaniec et al., 2001, Samnegard et al., 2001a, Samnegard et al., 2001b). In the present study, the use of ZOL following TPTD treatment increased the BMD of the proximal tibia by approximately 2.9% over 4 months (Fig. 6b), which is similar to the 2.3% increase in lumbar spine BMD over a 1-year period reported in a clinical study of the sequential use of once-weekly TPTD followed by a BP (Sugimoto et al., 2013). This indicates that ZOL effectively increases BMD after discontinuation of TPTD treatment in osteoporotic patients in a manner similar to that of other BPs.

To understand why in the present study the effects of TPTD were evident almost immediately after the switch from ZOL to TPTD treatment, detailed analyses of the changes in bone tissues and turnover immediately after the switch would be useful. Moreover, to elucidate whether our results were attributable to differences in the properties of ZOL in comparison to other BP agents or to the characteristic low dosing frequency of ZOL, further studies are needed that compare ZOL to other BP agents, and compare different ZOL dosing frequencies.

This study had several limitations. First, bone modeling in the rat differs from bone modeling in humans in that rats do not have Haversian canals, which limits their usefulness for investigations of cortical porosity. In osteoporotic patients, switching to daily TPTD after BP treatment has resulted in early decreases in BMD at the proximal femur. Although no cause for such a phenomenon has been identified, the reason may be that daily treatment with TPTD induces cortical porosity (Hirano et al., 1999, Burr et al., 2001, Hansen et al., 2013).To elucidate the cause of this phenomenon, it is probably necessary to conduct a study using human subjects or an animal model with cortical bones that undergo osteonal remodeling.

The results of our present study may have implications for clinical therapy of osteoporotic patients. Our findings suggest that switching from ZOL to TPTD treatment may effectively increase BMD in osteoporotic patients who have reached a BMD plateau with ZOL treatment alone. Sequential therapies are needed to effectively treat patients who respond poorly to initial treatment and patients with increasingly severe osteoporosis for whom their first therapy alone provides inadequate long-term improvements. Although quite a few clinicians have concerns regarding switching from BP treatment to TPTD, the results of our present study indicate that good results can be obtained with this sequential therapy.

At present, BPs are commonly recommended for follow-up treatment to prevent BMD reductions after termination of TPTD treatment; our results suggest that ZOL is a BP suitable for this use. In addition, ZOL treatment has been shown to have better patient compliance than other oral BP agents (Curtis et al., 2012). Thus, ZOL may be a suitable BP for maintenance therapy after withdrawal of TPTD treatment.

## 5 Conclusions

The present study showed that, in OVX rats, switching from ZOL to TPTD resulted in a non-attenuated anabolic response in the lumbar spine and femur. In addition, after the switch from TPTD to ZOL treatment, BMD was either maintained or further increased. If these results are reproduced in the clinical setting, the sequential use of ZOL followed by TPTD, or vice versa, in the treatment of osteoporosis patients would contribute to increases in BMD that would hopefully translate into a corresponding decrease in the incidence of vertebral and non-vertebral fractures.

## Appendix A Supplementary data

Supplementary tables Image 1

## References

[bb0005] Arlot M., Meunier P.J., Boivin G., Haddock L., Tamayo J., Correa-Rotter R., Jasqui S., Donley D.W., Dalsky G.P., Martin J.S., Eriksen E.F. (2005). Differential effects of teriparatide and alendronate on bone remodeling in postmenopausal women assessed by histomorphometric parameters. J. Bone Miner. Res..

[bb0010] Black D.M., Greenspan S.L., Ensrud K.E., Palermo L., McGowan J.A., Lang T.F., Garnero P., Bouxsein M.L., Bilezikian J.P., Rosen C.J. (2003). The effects of parathyroid hormone and alendronate alone or in combination in postmenopausal osteoporosis. N. Engl. J. Med..

[bb0030] Black D.M., Reid I.R., Boonen S., Bucci-Rechtweg C., Cauley J.A., Cosman F., Cummings S.R., Hue T.F., Lippuner K., Lakatos P., Leung P.C., Man Z., Martinez R.L., Tan M., Ruzycky M.E., Su G., Eastell R. (2010). The effect of 3 versus 6 years of zoledronic acid treatment of osteoporosis: a randomized extension to the HORIZON-Pivotal Fracture Trial (PFT). J. Bone Miner. Res..

[bb0015] Boonen S., Marin F., Obermayer-Pietsch B., Simoes M.E., Barker C., Glass E.V., Hadji P., Lyritis G., Oertel H., Nickelsen T., McCloskey E.V., Investigators E. (2008). Effects of previous antiresorptive therapy on the bone mineral density response to two years of teriparatide treatment in postmenopausal women with osteoporosis. J. Clin. Endocrinol. Metab..

[bb0020] Burr D.B., Hirano T., Turner C.H., Hotchkiss C., Brommage R., Hock J.M. (2001). Intermittently administered human parathyroid hormone(1-34) treatment increases intracortical bone turnover and porosity without reducing bone strength in the humerus of ovariectomized cynomolgus monkeys. J. Bone Miner. Res..

[bb0025] Chavassieux P.M., Arlot M.E., Reda C., Wei L., Yates A.J., Meunier P.J. (1997). Histomorphometric assessment of the long-term effects of alendronate on bone quality and remodeling in patients with osteoporosis. J. Clin. Invest..

[bb0035] Cosman F. (2014). Anabolic and antiresorptive therapy for osteoporosis: combination and sequential approaches. Curr. Osteoporos. Rep..

[bb0040] Cosman F., Eriksen E.F., Recknor C., Miller P.D., Guanabens N., Kasperk C., Papanastasiou P., Readie A., Rao H., Gasser J.A., Bucci-Rechtweg C., Boonen S. (2011). Effects of intravenous zoledronic acid plus subcutaneous teriparatide [rhPTH(1-34)] in postmenopausal osteoporosis. J. Bone Miner. Res..

[bb0045] Curtis J.R., Yun H., Matthews R., Saag K.G., Delzell E. (2012). Adherence with intravenous zoledronate and intravenous ibandronate in the United States Medicare population. Arthritis Care Res..

[bb0050] Ejersted C., Oxlund H., Eriksen E.F., Andreassen T.T. (1998). Withdrawal of parathyroid hormone treatment causes rapid resorption of newly formed vertebral cancellous and endocortical bone in old rats. Bone.

[bb0055] Ettinger B., San Martin J., Crans G., Pavo I. (2004). Differential effects of teriparatide on BMD after treatment with raloxifene or alendronate. J. Bone Miner. Res..

[bb0060] Finkelstein J.S., Hayes A., Hunzelman J.L., Wyland J.J., Lee H., Neer R.M. (2003). The effects of parathyroid hormone, alendronate, or both in men with osteoporosis. N. Engl. J. Med..

[bb0065] Finkelstein J.S., Wyland J.J., Lee H., Neer R.M. (2010). Effects of teriparatide, alendronate, or both in women with postmenopausal osteoporosis. J. Clin. Endocrinol. Metab..

[bb0070] Gasser J.A., Kneissel M., Thomsen J.S., Mosekilde L. (2000). PTH and interactions with bisphosphonates. J. Musculoskelet. Neuronal Interact..

[bb0075] Gasser J.A., Ingold P., Venturiere A., Shen V., Green J.R. (2008). Long-term protective effects of zoledronic acid on cancellous and cortical bone in the ovariectomized rat. J. Bone Miner. Res..

[bb0080] Hansen S., Hauge E.M., Beck Jensen J.E., Brixen K. (2013). Differing effects of PTH 1-34, PTH 1-84, and zoledronic acid on bone microarchitecture and estimated strength in postmenopausal women with osteoporosis: an 18-month open-labeled observational study using HR-pQCT. J. Bone Miner. Res..

[bb0085] Hirano T., Burr D.B., Turner C.H., Sato M., Cain R.L., Hock J.M. (1999). Anabolic effects of human biosynthetic parathyroid hormone fragment (1-34), LY333334, on remodeling and mechanical properties of cortical bone in rabbits. J. Bone Miner. Res..

[bb0090] Iwaniec U.T., Samnegard E., Cullen D.M., Kimmel D.B. (2001). Maintenance of cancellous bone in Ovariectomized, human parathyroid hormone [hPTH(1-84)]-treated rats by estrogen, risedronate, or reduced hPTH. Bone.

[bb0095] Kaufman J.M., Orwoll E., Goemaere S., San Martin J., Hossain A., Dalsky G.P., Lindsay R., Mitlak B.H. (2005). Teriparatide effects on vertebral fractures and bone mineral density in men with osteoporosis: treatment and discontinuation of therapy. Osteoporos. Int..

[bb0100] Kurland E.S., Heller S.L., Diamond B., McMahon D.J., Cosman F., Bilezikian J.P. (2004). The importance of bisphosphonate therapy in maintaining bone mass in men after therapy with teriparatide [human parathyroid hormone(1-34)]. Osteoporos. Int..

[bb0105] Ma Y.F., Li X.J., Jee W.S., McOsker J., Liang X.G., Setterberg R., Chow S.Y. (1995). Effects of prostaglandin E2 and F2 alpha on the skeleton of osteopenic ovariectomized rats. Bone.

[bb0110] Miki T., Nakatsuka K., Naka H., Masaki H., Imanishi Y., Ito M., Inaba M., Morii H., Nishizawa Y. (2004). Effect and safety of intermittent weekly administration of human parathyroid hormone 1-34 in patients with primary osteoporosis evaluated by histomorphometry and microstructural analysis of iliac trabecular bone before and after 1 year of treatment. J. Bone Miner. Metab..

[bb0115] Miller P.D., Delmas P.D., Lindsay R., Watts N.B., Luckey M., Adachi J., Saag K., Greenspan S.L., Seeman E., Boonen S., Meeves S., Lang T.F., Bilezikian J.P., I. Open-label Study to Determine How Prior Therapy with Alendronate or Risedronate in Postmenopausal Women with Osteoporosis Influences the Clinical Effectiveness of Teriparatide (2008). Early responsiveness of women with osteoporosis to teriparatide after therapy with alendronate or risedronate. J. Clin. Endocrinol. Metab..

[bb0120] Molster A.O. (1986). Biomechanical effects of intramedullary reaming and nailing on intact femora in rats. Clin. Orthop. Relat. Res..

[bb0125] Mosekilde L., Danielsen C.C., Knudsen U.B. (1993). The effect of aging and ovariectomy on the vertebral bone mass and biomechanical properties of mature rats. Bone.

[bb0130] Obermayer-Pietsch B.M., Marin F., McCloskey E.V., Hadji P., Farrerons J., Boonen S., Audran M., Barker C., Anastasilakis A.D., Fraser W.D., Nickelsen T., Investigators E. (2008). Effects of two years of daily teriparatide treatment on BMD in postmenopausal women with severe osteoporosis with and without prior antiresorptive treatment. J. Bone Miner. Res..

[bb0135] Parfitt A.M., Drezner M.K., Glorieux F.H., Kanis J.A., Malluche H., Meunier P.J., Ott S.M., Recker R.R. (1987). Bone histomorphometry: standardization of nomenclature, symbols, and units. Report of the ASBMR Histomorphometry Nomenclature Committee. J. Bone Miner. Res..

[bb0140] Prince R., Sipos A., Hossain A., Syversen U., Ish-Shalom S., Marcinowska E., Halse J., Lindsay R., Dalsky G.P., Mitlak B.H. (2005). Sustained nonvertebral fragility fracture risk reduction after discontinuation of teriparatide treatment. J. Bone Miner. Res..

[bb0145] Rhee Y., Won Y.Y., Baek M.H., Lim S.K. (2004). Maintenance of increased bone mass after recombinant human parathyroid hormone (1-84) with sequential zoledronate treatment in ovariectomized rats. J. Bone Miner. Res..

[bb0150] Samnegard E., Akhter M.P., Recker R.R. (2001). Maintenance of vertebral body bone mass and strength created by human parathyroid hormone treatment in ovariectomized rats. Bone.

[bb0155] Samnegard E., Iwaniec U.T., Cullen D.M., Kimmel D.B., Recker R.R. (2001). Maintenance of cortical bone in human parathyroid hormone(1-84)-treated ovariectomized rats. Bone.

[bb0160] Shahnazari M., Yao W., Wang B., Panganiban B., Ritchie R.O., Hagar Y., Lane N.E. (2011). Differential maintenance of cortical and cancellous bone strength following discontinuation of bone-active agents. J. Bone Miner. Res..

[bb0165] Sogaard C.H., Wronski T.J., McOsker J.E., Mosekilde L. (1994). The positive effect of parathyroid hormone on femoral neck bone strength in ovariectomized rats is more pronounced than that of estrogen or bisphosphonates. Endocrinology.

[bb0170] Sugie-Oya A., Takakura A., Takao-Kawabata R., Sano H., Shimazu Y., Isogai Y., Yamaguchi A., Ishizuya T. (2016). Comparison of treatment effects of teriparatide and the bisphosphonate risedronate in an aged, osteopenic, ovariectomized rat model under various clinical conditions. J. Bone Miner. Metab..

[bb0175] Sugimoto T., Shiraki M., Nakano T., Kishimoto H., Ito M., Fukunaga M., Hagino H., Sone T., Kuroda T., Nakamura T. (2013). Vertebral fracture risk after once-weekly teriparatide injections: follow-up study of Teriparatide Once-weekly Efficacy Research (TOWER) trial. Curr. Med. Res. Opin..

[bb0180] Sugimoto T., Nakamura T., Nakamura Y., Isogai Y., Shiraki M. (2014). Profile of changes in bone turnover markers during once-weekly teriparatide administration for 24 weeks in postmenopausal women with osteoporosis. Osteoporos. Int..

[bb0185] Takakura A., Takao-Kawabata R., Isogai Y., Kajiwara M., Murayama H., Ejiri S., Ishizuya T. (2016). Differences in vertebral, tibial, and iliac cancellous bone metabolism in ovariectomized rats. J. Bone Miner. Metab..

[bb0190] Vegger J.B., Nielsen E.S., Bruel A., Thomsen J.S. (2014). Additive effect of PTH (1-34) and zoledronate in the prevention of disuse osteopenia in rats. Bone.

[bb0195] Weiss H.M., Pfaar U., Schweitzer A., Wiegand H., Skerjanec A., Schran H. (2008). Biodistribution and plasma protein binding of zoledronic acid. Drug Metab. Dispos..

[bb0200] Yao W., Su M., Zhang Q., Tian X., Setterberg R.B., Blanton C., Lundy M.W., Phipps R., Jee W.S. (2007). Risedronate did not block the maximal anabolic effect of PTH in aged rats. Bone.

[bb0205] Zhou J., Wang T., Zhao X., Miller D.R., Zhai S. (2016). Comparative efficacy of bisphosphonates to prevent fracture in men with osteoporosis: a systematic review with network meta-analyses. Rheumatol. Ther.

