# Supplementary material for: Sequential treatment with zoledronic acid followed by teriparatide or vice versa increases bone mineral density and bone strength in ovariectomized rats
Source: Bone Rep. 2017 Aug 19;7:70–82. doi: 10.1016/j.bonr.2017.06.001 (PMC5602747; doi:10.1016/j.bonr.2017.06.001)
Supplement: Supplementary file 1 — Supplementary tables [file mmc1.docx]

Supplemental Table 1.

Detailed analysis of the bone mineral density changes at the proximal tibia in all treatment groups.

a: p<0.05, significant difference between Sham and V-V (Student’s T-test). b: p<0.05, significant difference vs V-V (Dunnett-test). S.D. = standard deviation

| *Change in BMD (%)* | | Sham (n=14) | V-V (n=19) | Z-V (n=17) | V-T (n=20) | Z-T (n=18) | T-V (n=20) | V-Z (n=18) | T-Z (n=18) |
| --- | --- | --- | --- | --- | --- | --- | --- | --- | --- |
| -2 month | mean | -0.3a | 12.1 | 13.0 | 13.0 | 14.6 | 14.9 | 14.6 | 13.3 |
|  | S.D. | 3.0 | 5.9 | 3.9 | 4.5 | 7.2 | 4.0 | 4.4 | 4.4 |
| 0 month | mean | 0.0 | 0.0 | 0.0 | 0.0 | 0.0 | 0.0 | 0.0 | 0.0 |
|  | S.D. | 0.0 | 0.0 | 0.0 | 0.0 | 0.0 | 0.0 | 0.0 | 0.0 |
| 1 month | mean | 1.1a | -3.5 | 4.1b | -3.5 | 5.3b | 1.9b | -2.5 | 4.0b |
|  | S.D. | 3.0 | 2.9 | 3.0 | 2.4 | 3.8 | 3.2 | 4.1 | 3.7 |
| 2 month | mean | 0.3a | -5.6 | 5.9b | -5.5 | 6.7b | 4.5b | -3.9 | 5.1b |
|  | S.D. | 4.0 | 4.4 | 2.7 | 3.2 | 3.7 | 4.0 | 3.7 | 4.5 |
| 4 month | mean | 0.7a | -8.0 | 7.6b | -7.3 | 9.2b | 4.9b | -7.5 | 7.1b |
|  | S.D. | 4.4 | 5.3 | 3.4 | 5.2 | 4.0 | 6.2 | 5.0 | 5.4 |
| 5 month | mean | -1.7a | -9.2 | 7.9b | -4.7 | 12.1b | -2.9b | -5.8 | 9.1b |
|  | S.D. | 5.1 | 6.3 | 3.0 | 6.3 | 4.6 | 7.5 | 7.1 | 5.9 |
| 6 month | mean | -1.3a | -9.6 | 8.8b | 0.3b | 14.3b | -5.6 | -2.2b | 9.8b |
|  | S.D. | 5.4 | 6.5 | 5.1 | 6.7 | 5.9 | 9.0 | 5.6 | 8.1 |
| 8 month | mean | -4.9a | -12.5 | 9.1b | 0.5b | 16.2b | -11.1 | -2.2b | 10.2b |
|  | S.D. | 6.0 | 6.5 | 3.4 | 7.9 | 6.0 | 7.4 | 6.3 | 6.6 |

Supplemental Table 2.

Detailed analysis of the markers of bone metabolism in all treatment groups.

a: p<0.05, significant difference between Sham and V-V (Student’s T-test). b: p<0.05, significant difference vs V-V (Dunnett-test). S.D. = standard deviation

| *Serum osteocalcin (ng/mL)* | | Sham (n=14) | V-V (n=19) | Z-V (n=17) | V-T (n=20) | Z-T (n=18) | T-V (n=20) | V-Z (n=18) | T-Z (n=18) |
| --- | --- | --- | --- | --- | --- | --- | --- | --- | --- |
| 0 month | mean | 17.8a | 23.3 | 24.9 | 22.6 | 26.7 | 22.5 | 24.2 | 22.3 |
|  | S.D. | 2.9 | 4.3 | 3.9 | 3.4 | 4.3 | 3.8 | 5.3 | 4.4 |
| 1 month | mean | 16.3a | 20.8 | 14.6b | 21.6 | 15.1b | 24.4b | 20.8 | 25.1b |
|  | S.D. | 3.2 | 4.9 | 3.2 | 4.0 | 3.9 | 4.2 | 3.3 | 4.9 |
| 2 month | mean | 17.2a | 22.9 | 14.3b | 23.4 | 13.9b | 25.6 | 23.3 | 26.3 |
|  | S.D. | 3.4 | 5.4 | 3.8 | 5.1 | 2.9 | 6.2 | 6.3 | 4.4 |
| 4 month | mean | 11.2a | 16.9 | 9.8b | 15.9 | 10.2b | 20.3 | 17.1 | 19.9 |
|  | S.D. | 2.5 | 4.9 | 3.3 | 4.9 | 2.6 | 6.2 | 5.9 | 5.0 |
| 5 month | mean | 13.8a | 19.0 | 12.8b | 22.5b | 18.2 | 17.3 | 13.3b | 11.4b |
|  | S.D. | 3.3 | 4.2 | 2.5 | 4.5 | 4.0 | 3.2 | 3.9 | 2.5 |
| 6 month | mean | 15.0a | 20.9 | 13.2b | 22.7 | 19.7 | 19.0 | 14.0b | 11.2b |
|  | S.D. | 3.2 | 3.3 | 3.0 | 4.8 | 4.1 | 3.7 | 3.4 | 3.1 |
| 8 month | mean | 15.2a | 21.8 | 14.5b | 25.8b | 20.7 | 21.4 | 14.2b | 12.8b |
|  | S.D. | 2.5 | 5.2 | 3.4 | 6.0 | 5.1 | 6.0 | 3.5 | 1.8 |
|  | |  |  |  |  |  |  |  |  |
| *Urine CTX (ng/mg∙Cr)* | | Sham (n=14) | V-V (n=19) | Z-V (n=17) | V-T (n=20) | Z-T (n=18) | T-V (n=20) | V-Z (n=18) | T-Z (n=18) |
| 0 month | mean | 12.2a | 22.9 | 20.6 | 25.1 | 22.1 | 21.0 | 21.4 | 21.3 |
|  | S.D. | 7.9 | 8.3 | 3.6 | 6.3 | 7.9 | 5.8 | 7.4 | 6.4 |
| 1 month | mean | 8.5a | 16.5 | 3.0b | 16.4 | 3.2b | 15.0 | 14.2 | 13.6 |
|  | S.D. | 3.0 | 4.7 | 0.8 | 3.3 | 1.1 | 3.6 | 3.6 | 3.9 |
| 2 month | mean | 8.0a | 15.3 | 3.7b | 15.4 | 3.4b | 11.5b | 13.2 | 11.0b |
|  | S.D. | 2.3 | 4.3 | 1.1 | 4.9 | 1.3 | 2.5 | 3.1 | 3.4 |
| 4 month | mean | 7.1a | 13.9 | 3.3b | 12.0 | 3.4b | 10.4b | 10.3b | 10.0b |
|  | S.D. | 2.1 | 5.6 | 0.9 | 3.9 | 0.8 | 3.3 | 3.9 | 3.4 |
| 5 month | mean | 9.3a | 16.4 | 4.6b | 15.4 | 5.1b | 16.4 | 2.8b | 2.5b |
|  | S.D. | 3.9 | 6.7 | 1.6 | 4.5 | 1.6 | 6.1 | 1.6 | 0.9 |
| 6 month | mean | 8.6a | 14.2 | 4.9b | 12.4 | 5.1b | 15.7 | 2.8b | 2.6b |
|  | S.D. | 3.1 | 5.7 | 1.5 | 4.2 | 2.1 | 4.8 | 1.1 | 1.0 |
| 8 month | mean | 8.3a | 14.1 | 5.5b | 13.0 | 5.8b | 15.9 | 3.8b | 3.6b |
|  | S.D. | 4.0 | 6.6 | 1.6 | 6.6 | 2.0 | 5.4 | 1.4 | 1.1 |

Supplemental Table.3 :

Detailed analysis of the results of histomorphometry of the L5 vertebra for all groups.

a: p<0.05, significant difference between Sham and V-V (Student’s T-test). b: p<0.05, significant difference vs V-V (Dunnett-test). c: p<0.05, significant difference between the Z-T group and Z-V group. d: p<0.05, significant difference between the T-Z group and T-V group. S.D. = standard deviation

|  |  | Sham n=6 | V-V  n=6 | Z-V  n=6 | V-T  n=6 | Z-T  n=6 | T-V  n=6 | V-Z  n=6 | T-Z  n=6 |
| --- | --- | --- | --- | --- | --- | --- | --- | --- | --- |
| *Structural parameter* | | | | | | | | | |
| BV/TV (%) | Mean | 29.32a | 21.17 | 29.14 | 28.89 | 42.95b,c | 24.05 | 23.18 | 37.04b,d |
|  | S.D. | 6.14 | 5.97 | 6.45 | 8.92 | 7.31 | 8.92 | 7.24 | 9.17 |
| Tb.Th (µm) | Mean | 63.90 | 72.43 | 78.21 | 94.98 | 107.17b,c | 68.89 | 74.83 | 94.55d |
|  | S.D. | 9.18 | 16.08 | 11.41 | 24.45 | 12.65 | 12.97 | 10.19 | 16.03 |
| Tb.N (N/mm) | Mean | 4.56a | 2.91 | 3.72 | 3.03 | 3.99b | 3.40 | 3.04 | 3.90b |
|  | S.D. | 0.51 | 0.63 | 0.55 | 0.57 | 0.32 | 0.72 | 0.69 | 0.47 |
| *Formation parameter* | | | | | | | | | |
| OS/BS (%) | Mean | 7.12a | 21.58 | 2.01b | 34.78b | 8.75b,c | 29.87 | 4.80b | 2.49b,d |
|  | S.D. | 2.81 | 10.21 | 1.84 | 9.22 | 2.10 | 6.85 | 4.08 | 1.75 |
| Ob.S/BS (%) | Mean | 5.12a | 11.89 | 3.25b | 20.33b | 8.98c | 17.12b | 5.08b | 3.83b,d |
|  | S.D. | 1.98 | 4.48 | 2.03 | 3.18 | 2.30 | 2.22 | 3.22 | 1.60 |
| dLS/BS (%) | Mean | 2.06 | 4.74 | 0.02 | 15.16b | 2.66c | 12.45b | 0.29 | 0.00d |
|  | S.D. | 1.60 | 3.33 | 0.06 | 7.45 | 1.67 | 7.12 | 0.54 | 0.00 |
| sLS/BS (%) | Mean | 20.55a | 32.33 | 14.11b | 35.23 | 22.83b,c | 37.73 | 14.19b | 14.30b,d |
|  | S.D. | 4.72 | 8.42 | 5.90 | 4.62 | 3.85 | 3.46 | 4.06 | 5.93 |
| MS/BS (%) | Mean | 12.34a | 20.91 | 7.08b | 32.78b | 14.07c | 31.32b | 7.39b | 7.15b,d |
|  | S.D. | 3.73 | 5.49 | 2.98 | 6.18 | 3.05 | 7.26 | 2.35 | 2.97 |
| BFR/BV (%/year) | Mean | 85.85 | 130.14 | 5.60b | 187.22 | 43.25c | 263.85b | 17.02b | 0.00b,d |
|  | S.D. | 50.90 | 51.21 | 13.72 | 113.08 | 11.40 | 111.34 | 29.23 | 0.00 |
| *Resorption parameter* | | | | | | | | | |
| ES/BS (%) | Mean | 13.46 | 18.97 | 6.17b | 16.55 | 8.58b | 19.40 | 9.55b | 7.26b,d |
|  | S.D. | 3.93 | 5.78 | 2.26 | 3.92 | 3.42 | 1.71 | 3.10 | 2.59 |
| Oc.S/BS (%) | Mean | 3.64 | 6.09 | 2.18b | 8.61 | 3.41 | 7.61 | 3.40 | 2.69b,d |
|  | S.D. | 1.91 | 2.27 | 1.29 | 2.62 | 2.07 | 1.39 | 1.18 | 1.46 |
